# Hydrothermal Corrosion of Latest Generation of FeCrAl Alloys for Nuclear Fuel Cladding

**DOI:** 10.3390/ma17071633

**Published:** 2024-04-03

**Authors:** Bhavani Sasank Nagothi, Haozheng Qu, Wanming Zhang, Rajnikant V. Umretiya, Evan Dolley, Raul B. Rebak

**Affiliations:** 1 General Electric Research Center, 1 Research Circle, Schenectady, NY 12309, USA; 2 College of Nanotechnology, Science, and Engineering, University at Albany, Albany, NY 12203, USA

**Keywords:** FA-SMT (Ferritic Alloy-Sandvik Material Technology), ATF (accident tolerant fuel), PM-C26M, hydrothermal corrosion, FeCrAl

## Abstract

After the Fukushima nuclear disaster, the nuclear materials community has been vastly investing in accident tolerant fuel (ATF) concepts to modify/replace Zircaloy cladding material. Iron–chromium–aluminum (FeCrAl) alloys are one of the leading contenders in this race. In this study, we investigated FA-SMT (or APMT-2), PM-C26M, and Fe17Cr5.5Al over a time period of 6 months in simulated BWR environments and compared their performance with standard Zirc-2 and SS316 materials. Our results implied that water chemistry along with alloy chemistry has a profound effect on the corrosion rate of FeCrAl alloys. Apart from SS316 and Zirc-2 tube specimens, all FeCrAl alloys showed a mass loss in hydrogen water chemistry (HWC). FA-SMT displayed minimal mass loss compared to PM-C26M and Fe17Cr5.5Al because of its higher Cr content. The mass gain of FeCrAl alloys in normal water chemistry (NWC) is significantly less when compared to Zirc-2.

## 1. Introduction

A continued demand to generate electricity while mitigating greenhouse gas emissions is possible to fulfill through nuclear power plants [1]. In all Light Water Reactors (LWRs), fuel pellets are protected in cladding tubes. Cladding material (zirconium-based alloys) plays a crucial role as a barrier by preventing the release of fuel into the coolant and by protecting the fuel from the coolant’s corrosion [2]. Under normal operating conditions, using Zr-based alloys as fuel cladding material is a good choice due to their low thermal neutron absorption [3] and their acceptable mechanical properties under irradiation [4]. The problem arises in the absence of a coolant, leading to the meltdown of the core and ultimately resulting in a loss of coolant accident (LOCA) (Fukushima nuclear disaster) [5]. Thus, there is an urgent need for the development of accident tolerant fuels (ATFs).

ATF cladding designs should survive LOCA scenarios; hence, they should be resistant to oxidation at high temperatures [6] and have a good corrosion performance under normal operating conditions [7]. Currently, materials are being explored in two different approaches to meet the ATF cladding needs. Coated zirconium alloys are being used as a short-term approach, along with monolithic FeCrAl alloys, and silicon carbide composites are being used as a mid-term approach [8,9]. Among these concepts, FeCrAl alloys have exhibited superior corrosion resistance in operational conditions [10,11] and LOCA conditions [12] and can even prevent detrimental fuel/cladding chemical interaction [13]. The enhanced corrosion resistance of FeCrAl alloys comes from the formation of a Cr passivation layer, which will inhibit the corrosion and act as a protective layer [14]. In the mid-term approach, FeCrAl alloys may replace Zircaloy as cladding material. Thus, it is important to expose variants of FeCrAl alloys to simulated BWR-NWC (normal water chemistry) and BWR-HWC (hydrogen water chemistry) operating conditions to investigate the formation of a protective oxide on these alloys as a function of water chemistry.

To examine the corrosion behavior of FeCrAl alloy variants under reactor operating conditions, numerous studies were performed. In the case of first-generation FeCrAl alloys [11], APMT (21Cr) was studied extensively, and it displayed excellent corrosion resistance under high temperature (~300 °C) waters but was susceptible to radiation-induced embrittlement due to α’ precipitates of chromium [15,16]. Hence, for second-generation FeCrAl alloys [10], the Cr content was reduced to 12 wt%, and minor alloying elements were added (Y, Nb, Mo, and Si) to provide mechanical strengthening to these cladding tubes [17].

To study the impact of the microstructure, Umretiya et al. [18] performed a systematic study to understand the effects of the microstructure, manufacturing route, and composition on the corrosion behavior of FeCrAl alloys. They concluded that FeCrAl alloy hydrothermal corrosion resistance has little/less sensitivity to (microstructural) the fabrication route. At the same time, Yin et al. conducted a long-term immersion test [19], where they investigated the hydrothermal corrosion behavior of FeCrAl variants of APMT (21Cr) and wrought C-26M (12Cr) for over 12 months. Their results concluded that the corrosion behavior of wrought C-26M (12 Cr) was compromised when compared to APMT (21 Cr).

Hence, in this article, we emphasize our findings on the hydrothermal corrosion behavior of the three latest variants of FeCrAl alloys along with Zirc-2 and SS316. APMT was slightly modified as APMT-2 (FA-SMT); unlike the traditional wrought method, powder metallurgy was used to fabricate FA-SMT and C26M alloys. Along with the modified versions of first- and second-generation FeCrAl alloys, we also tested an in-between composition space of 17 Cr alloy (Fe17Cr5.5Al) to gain insights into the corrosion performance of this variant along with standard FeCrAl alloys. The immersion test was carried out for six months in a typical BWR environment under two simulated water chemistries at 288 °C. This particular work is a continuation of our previous study, where the preliminary data of this work were published by Rebak et al. [20]. In the current article, we are primarily focusing on the detailed characterization of the oxide layer that was developed as a function of water chemistry via a Transmission Electron Microscopy (TEM) analysis.

## 2. Experimental Conditions

As listed in Table 1, the three latest FeCrAl alloy variants along with the current nuclear cladding materials, Zirc-2 and SS316 (for reference), were tested for a time span of six months in simulated BWR environments. Apart from Fe17Cr5.5Al (flat coupon), the rest of all of the test samples were kept in tubes. The thickness of the powdered metallurgy-processed FA-SMT and PM-C26M tube walls was targeted to be 0.3 mm. The wall thickness of the Zirc-2 specimen, including the inner diameter liner, was targeted to be around 0.675 mm. All of the tube specimens that participated in the immersion testing were nominally 12.7 mm (0.5″) long sections of actual tube claddings with an OD of 10.26 mm. By using vacuum induction melting (VIM), model alloy Fe17Cr5.5Al was cast in rod shape. Flat coupons were cut from the rod-shaped master alloy to obtain a coupon with net exposed area of 500 mm^2^ with a hole to hang the coupons (1.5 mm diameter). By using 600-grit SiC paper, all samples were polished and later cleaned with Liquinox, isopropyl alcohol (IPA), and ultra-high-purity (UHP) water prior to corrosion testing.

Testing was carried out on 16 samples of each tubed alloy along with 6 samples of Fe17Cr5.5Al flat coupons. The testing conditions are shown in Table 2. By using UHP water of 18 Mega-Ohm (MΩ), testing was performed in a recirculating autoclave simulating the temperatures of BWR under two different water chemistries (HWC and NWC) with no addition of impurities. Hydrothermal corrosion testing was performed in separate autoclaves, which were circulating at a rate of 200 cm^3^/min. By using the right amount of gas, high-purity water was reconditioned in a 4 L glass column followed by pumping the water to autoclave using a pressure pump (high). Later, by using a back-pressure regulator, the pressure was controlled at approximately 10 MPa. The water conductivity was measured before entering and after exiting the autoclave. Samples were taken out after six months of immersion testing, rinsed with UHP water, and dried in ambient atmosphere. The mass change of each specimen was calculated three times and reported in mg/dm^2^. Mass gain during the autoclave exposure is shown in positive values, whereas negative values indicate a mass loss during the exposure.

After the water immersion, surface oxide characterization was performed using TEM. Using focused ion beam (FIB) milling, TEM lamellae were prepared for all samples. During FIB milling, a thin layer of Pt coating was applied to protect the corroded surface of the tested samples. At 300 kV, using a Thermo Scientific (Waltham, MA, USA) Themis Z aberration-corrected S/TEM, scanning transmission electron microscope (STEM), energy-dispersive X-ray spectroscopy (EDS) analysis, and bright-field (BF) TEM imaging were performed. TEM Instrument Analysis (TIA) software was used for image and composition analysis.

## 3. Results

For immersion corrosion testing, the guidance in the ASTM standards G1 and G31 [21] was followed. The experiments were designed in the vicinity of 300 °C [11,19] to study the plausible ATF candidate FeCrAl along with nuclear alloys.

**Figure 1:**
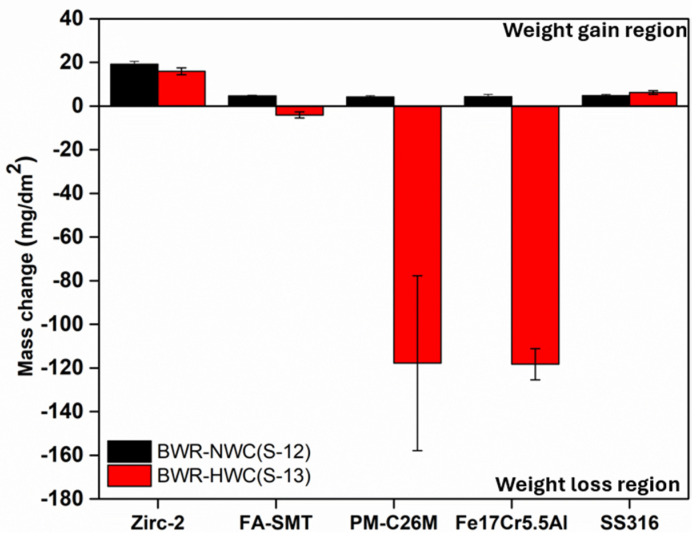
After six months of immersion testing, mass changes in FA-SMT, PM-C26M, SS316, and Zirc-2 tube specimens along with Fe17Cr5.5Al flat coupon specimens in simulated BWR-HWC and BWR-NWC waters (Positive value refers to wight gain, whereas negative value represents weight loss).

After six months of immersion testing, all 16 tubes of each alloy, along with six flat coupons of Fe17Cr5.5Al, were taken out to determine the mass change, as shown in Figure 1. Each individual sample was measured three times. All of the samples, irrespective of their compositions, displayed a mass gain under BWR-NWC (S-12) that contained 0.5 ppm of dissolved oxygen. Under these autoclave testing conditions, the Zirc-2 tube samples displayed a relatively higher mass gain (22 mg/dm^2^) than the FeCrAl alloys, and this observation is consistent with the literature [20]. The lower mass gain of the FeCrAl alloys could be due to the formation of a protective surface oxide. It is also observed that within the FeCrAl alloys, a slightly higher mass gain was observed in FA-SMT (5 mg/dm^2^) when compared to PM-C26M (4.5 mg/dm^2^), which may be due to the higher Cr content. The flat coupon of Fe17Cr5.5Al recorded a mass gain of 4.9 mg/dm^2^. However, in the BWR-HWC (S-13), PM-C26M and Fe17Cr5.5Al displayed a significant mass loss when compared to FA-SMT. The lower Cr content in Fe17Cr5.5Al and PM-C26M could be the reason for the mass loss in these alloys. The mass gain of the Zirc-2 tube specimens in BWR-HWC is relatively less when compared to BWR-NWC. A reducing (hydrogen) environment hindering the formation of a protective passive oxide layer could be the reason for this observation.

**Figure 2:**
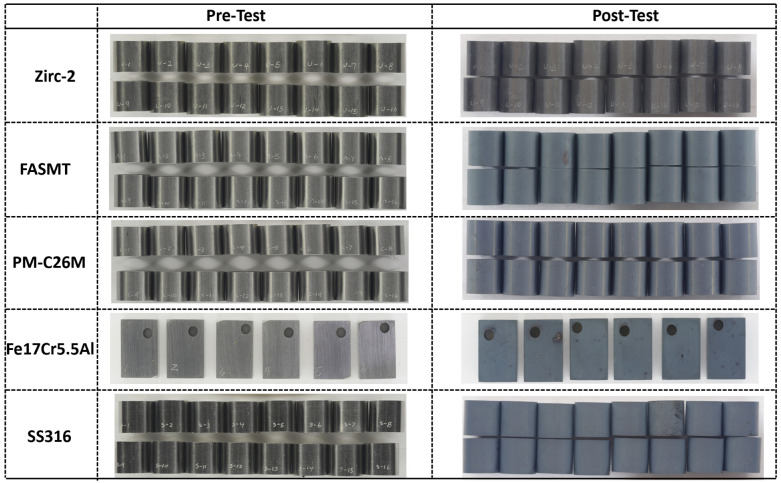
Visual inspection of Zirc-2, FA-SMT, PM-C26M, and SS316 tubes along with Fe17Cr5.5Al flat coupons after 6 months of immersion testing in BWR-NWC (S-12).

An optical inspection of the BWR-NWC test specimen after six months of immersion testing is shown in Figure 2. All of the test specimens exposed to the S-12 system (oxidizing) displayed a mass gain, as shown in Figure 1. The Zirc-2 tube specimens exposed to oxygenated waters developed black oxide, indicating the formation of protective ZrO_2_. The FA-SMT, PM-C26M, and Fe17Cr5.5Al specimens visually had a distant color compared to the Zirc-2 tube specimens. The FA-SMT specimens displayed a slightly different appearance than the PM-C26M and Fe17Cr5.5Al specimens, which could be related to the different surface corrosion products.

**Figure 3:**
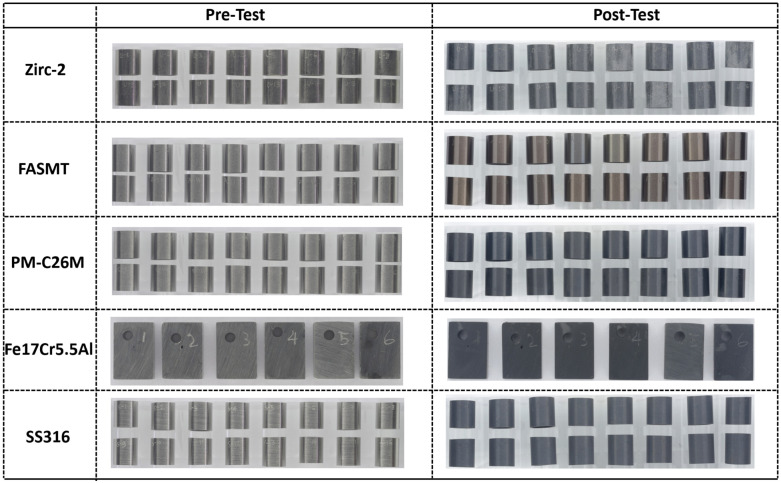
Visual inspection of Zirc-2, FA-SMT, PM-C26M, and SS316 tubes along with Fe17Cr5.5Al flat coupons after 6 months of immersion testing in BWR-HWC (S-13).

The specimens tested in BWR-HWC (S-13) are shown in Figure 3. In the hydrogenated waters, apart from the Zirc-2 and SS316 specimens, all of the FeCrAl variants showcased a mass loss. The Zirc-2 tubes tested in S-13 had a dull gray appearance. Unlike the FeCrAl alloy variants tested in S-12, the FeCrAl specimens tested in the S-13 system had a distinctive visual appearance. A shiny golden appearance for FA-SMT and a shiny gray appearance for PM-C26M were observed for the tube specimens tested in S-13, which could be due to a higher Cr content in FA-SMT than in PM-C26M.

After the mass change measurements and optical inspection, one of each FeCrAl alloy variant along with the Zirc-2 and SS316 tube specimens were underwent FIB for the TEM analysis.

### 3.1. TEM Analysis of Alloys Tested in BWR-NWC Environment

#### 3.1.1. Zirc-2 Tube

**Figure 4:**
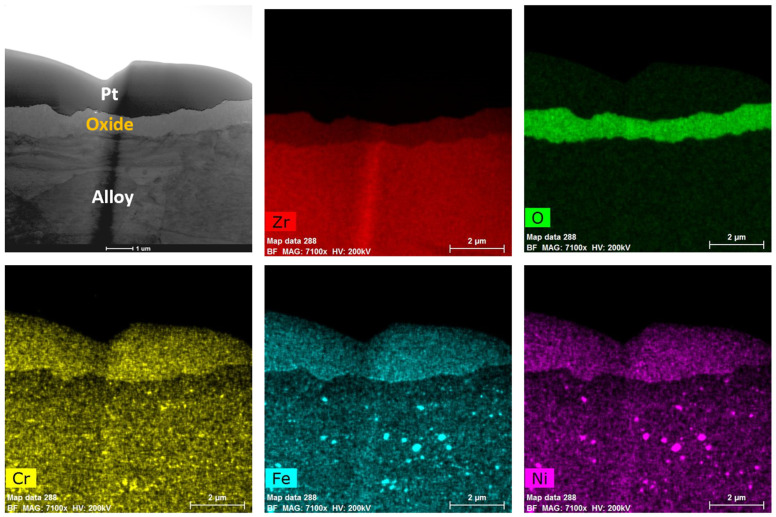
Low-magnification TEM-EDS for Zirc-2 (Tube) after 6 months of exposure in BWR-NWC (S-12). Marker is 2000 nm long.

**Figure 5:**
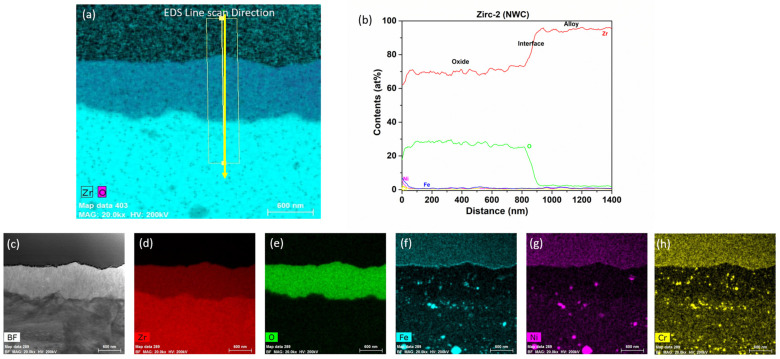
(**a**) shows the EDS line scan direction, (**b**) shows the line scans of the oxide–substrate interfaces in the Zirc-2 tube after 6 months of simulated BWR-NWC exposure, and (**c**–**h**) show the high magnification EDS mapping of the Zirc-2 tube.

A TEM analysis of the Zirc-2 (tube) after 6 months of immersion in the BWR-NWC (S-12) is shown in Figure 4. The oxide developed on the Zirc-2 tube looks almost uniform with no signs of nodular corrosion and is ~750 nm (Figure 5). A high-magnification TEM-EDS analysis confirmed the developed oxide to be ZrO_2_ with second-phase particles embedded in it (Fe, Cr, and Ni). It is also observed that the SPPs are distributed in the Zr substrate along with the developed oxide [22]. The Zirc-2 tube displayed a higher mass gain and developed a thicker external protective oxide layer when compared to all FeCrAl alloy variants under BWR-NWC.

#### 3.1.2. FA-SMT Tube

**Figure 6:**
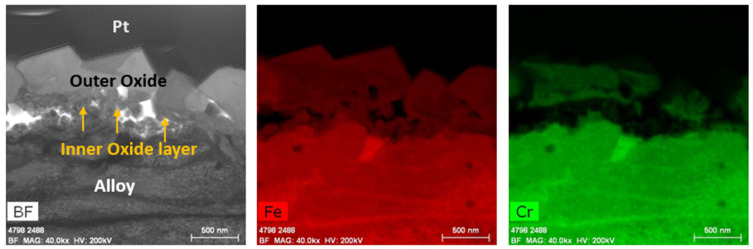

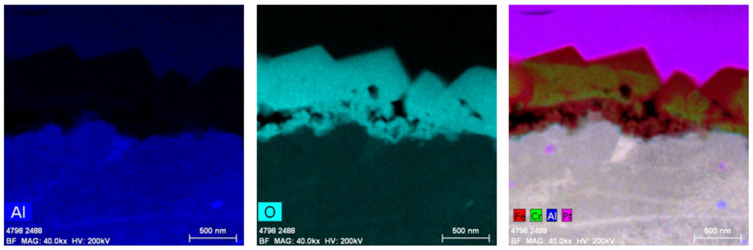
TEM-EDS for FA-SMT (tube) after 6 months of exposure in BWR-NWC (S-12). Marker is 500 nm long.

**Figure 7:**
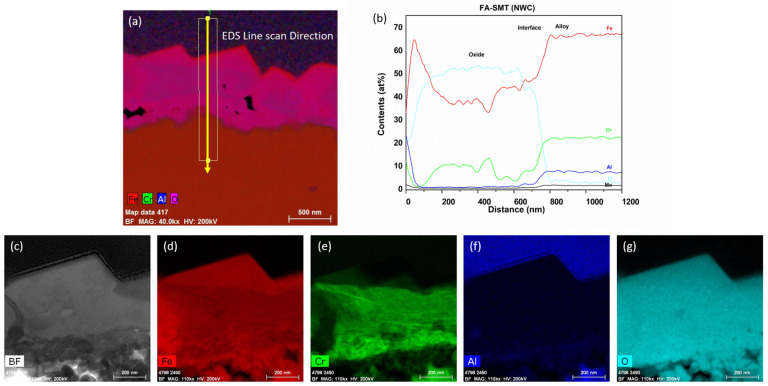
(**a**) shows the EDS line scan direction, (**b**) shows the line scans of the oxide–substrate interfaces in the FA-SMT tube after 6 months of simulated BWR-NWC exposure, and (**c**–**g**) show the high-magnification EDS mapping of the FA-SMT tube.

After immersion testing in the BWR-NWC (S-12) for six months, a TEM analysis was performed on one of the 16 FA-SMT tube specimens. The oxide layer thickness on the FA-SMT tube was calculated to be ~500 nm using a TEM bright field image (Figure 6). Low-magnification TEM-EDS elemental maps showcased a fine-grained internal layer at the oxide–alloy matrix along with a continuous thick external Fe-Cr spinel oxide. The fine-grained internal layer was mainly composed of Fe and Cr. As shown in Figure 7, no aluminum enrichment was observed to form beneath the surface oxide layers, as confirmed by the line scans and high magnification TEM-EDS maps, which is contradictory to the literature [10]. Even though the Fe-Cr-rich spinel oxide acts as a top-layer barrier between the water and substrate to allow for Cr oxide growth, the Cr oxide is non-uniform and defective. As shown in Figure 1, mass gain of the FA-SMT tube under this testing condition is due to the oxide formation as it limits the mass loss.

#### 3.1.3. PM-C26M Tube

**Figure 8:**
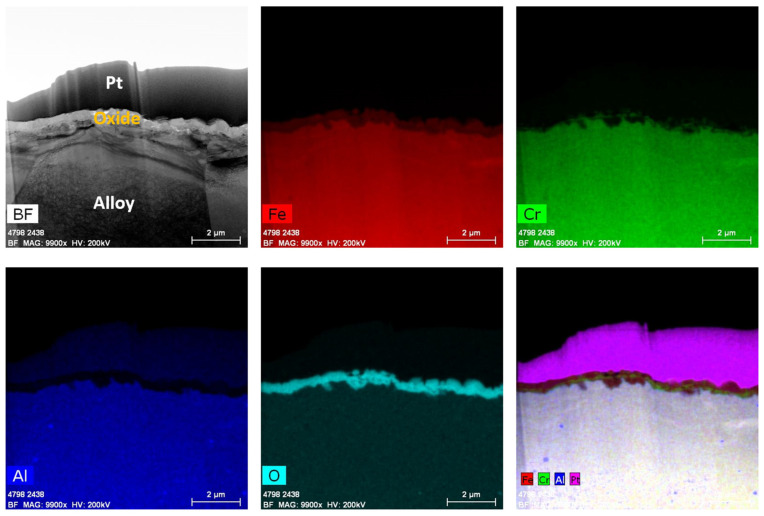
TEM-EDS for PM-C26M (tube) after 6 months of exposure in BWR-NWC (S-12). Marker is 2000 nm long.

**Figure 9:**
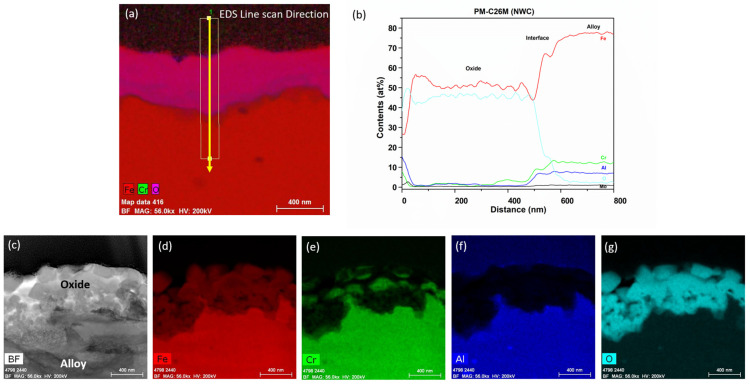
(**a**) shows the EDS line scan direction, (**b**) shows the line scans of the oxide–substrate interfaces in the PM-C26M tube after 6 months of simulated BWR-NWC exposure, and (**c**–**g**) show the high-magnification EDS mapping of the PM-C26M tube.

Figure 8 shows the oxide at ~450 nm that was developed on the PM-C26M tube after 6 months of BWR-NWC (S-12) exposure. In the EDS maps in Figure 9, it can be seen that the external oxide layer mainly consists of Cr oxide and Fe oxide, respectively. The oxide layer developed externally on PM-C26M is not continuous as Cr oxide regions are embedded in the surface Fe oxide layer. Due to this non-continuous protective Cr oxide layer, no enrichment of aluminum oxide is observed at the oxide–substrate interface (Figure 9). In the current test, the weight gain of PM-C26M was slightly lower than that of FA-SMT.

#### 3.1.4. Fe17Cr5.5Al Flat Coupon

**Figure 10:**
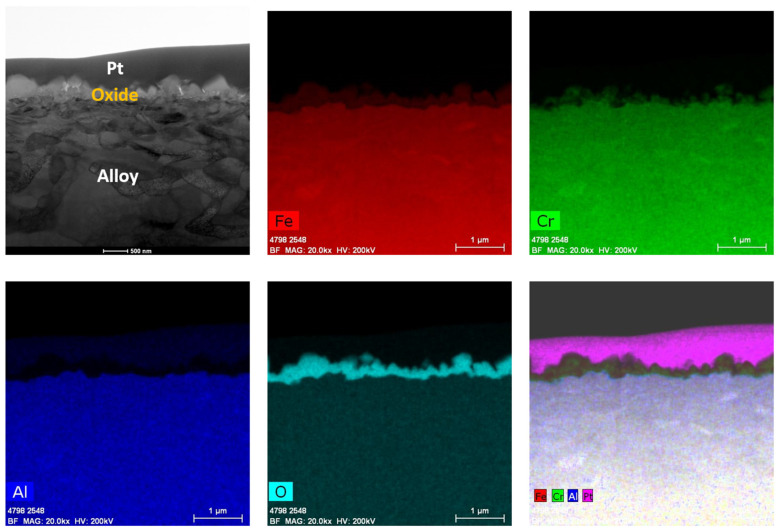
TEM-EDS for Fe17Cr5.5Al flat coupon after 6 months of exposure in BWR-NWC (S-12). Marker is 1000 nm long.

**Figure 11:**
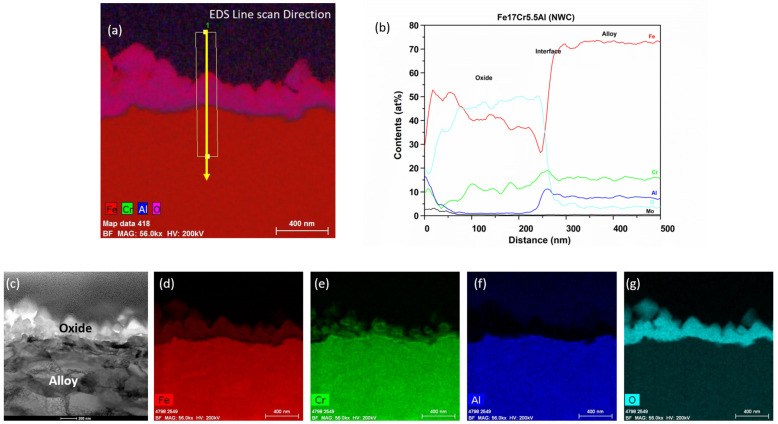
(**a**) shows the EDS line scan direction, (**b**) shows the line scans of the oxide–substrate interfaces in the Fe17Cr5.5Al flat coupon after 6 months of simulated BWR-NWC exposure, and (**c**–**g**) show the high-magnification EDS mapping of the Fe17Cr.5.5Al flat coupon.

For Fe17Cr5.5Al (flat coupons), the oxide thickness after six months of testing is ~230 nm (Figure 10). A three-layered oxide structure is showcased by the TEM-EDS maps in Figure 11. A thick FeCr-rich oxide top layer is followed by a continuous chromium-rich oxide layer. The aluminum layer formed underneath the Cr-rich oxide layer is not continuous and can be seen as an internal nodular oxide. The line scan in Figure 11 also confirms the enrichment of Al and Cr at the oxide–substrate interface. The enrichment of Al is proposed to be due to the low oxygen and partial pressure below the Cr oxide layer and above the substrate [23]. It should also be noted that the oxide developed on Fe17Cr5.5Al (flat coupon) is thinnest of all the FeCrAl alloys tested in this study.

#### 3.1.5. SS316 Tube

**Figure 12:**
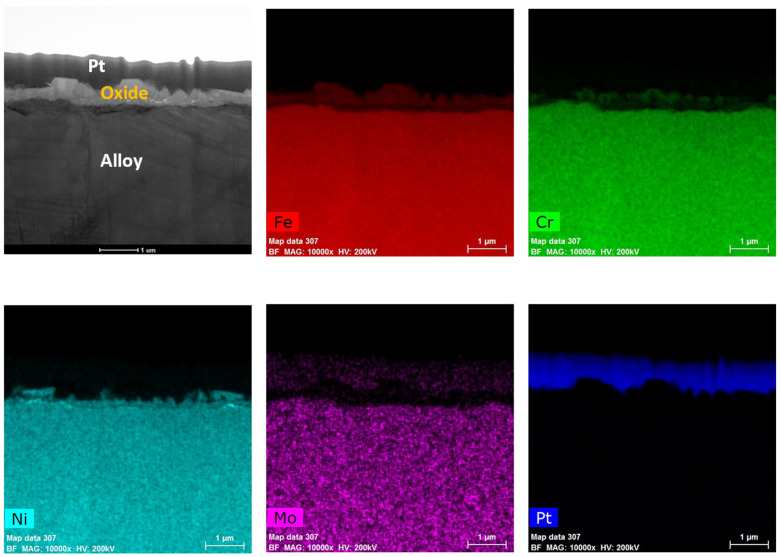
TEM-EDS for SS316 (tube) after 6 months of exposure in BWR-NWC (S-12). Marker is 1000 nm long.

**Figure 13:**
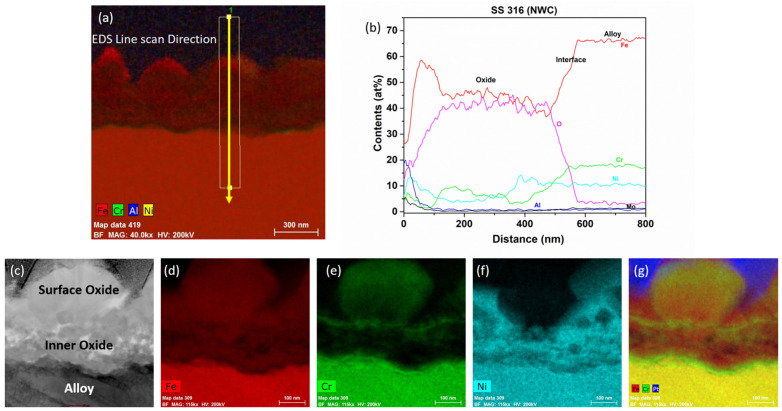
(**a**) shows the EDS line scan direction, (**b**) shows the line scans of the oxide–substrate interfaces in the SS316 tube after 6 months of simulated BWR-NWC exposure, and (**c**–**g**) show the high-magnification EDS mapping of the SS316 tube.

After immersion testing in the BWR-NWC (S-12) for six months, a TEM analysis was conducted on the SS316 tube specimens. The oxide layer formed on the SS316 tube was calculated to be ~500 nm using a TEM bright field image (Figure 12). As shown in Figure 13, the high-magnification TEM-EDS elemental maps showcase a surface oxide spinel along with a fine-grained inner oxide layer at the oxide–alloy matrix interface. The surface oxide mainly consists of an Fe-Cr spinel, whereas the fine-grained internal layer is composed of Fe, Cr, and Ni. Under oxygenated water, the SS316 tube specimens displayed a lower mass gain than the Zirc-2 tube specimens.

### 3.2. TEM Analysis of Alloys Tested in BWR-HWC Environment

#### 3.2.1. Zirc-2 Tube

**Figure 14:**
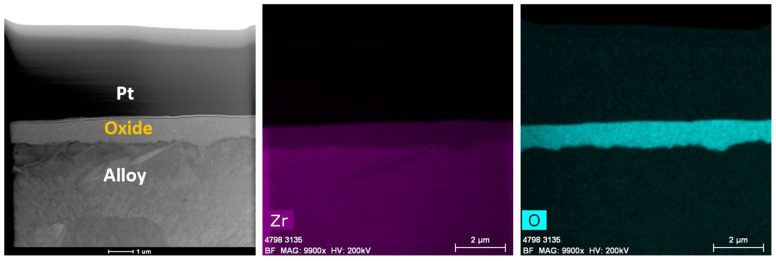

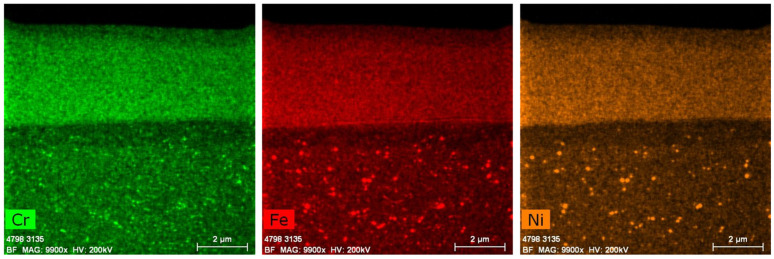
Low-magnification TEM-EDS images for Zirc-2 (Tube) after 6 months of exposure in BWR-HWC (S-13). Marker is 2000 nm long.

**Figure 15:**
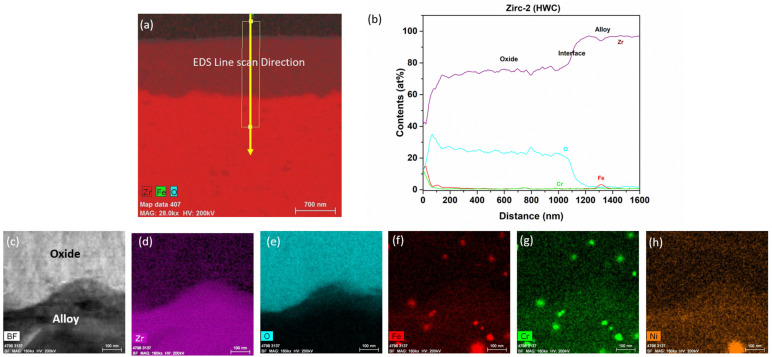
(**a**) shows the EDS line scan direction, (**b**) shows the line scans of the oxide–substrate interfaces in the Zirc-2 tube after 6 months of simulated BWR-HWC exposure, and (**c**–**h**) show the high-magnification EDS mapping of the Zirc-2 tube.

The TEM analysis of the Zirc-2 tube after 6 months of immersion in the BWR-HWC is shown in Figure 14. The oxide developed on the Zirc-2 tube looks almost uniform and is ~900 nm. A high-magnification TEM-EDS analysis (Figure 15) confirmed the developed oxide to be ZrO_2_ with SPP distributed in the Zr substrate along with the oxide [22]. Even though the thickness of the oxide developed on the Zirc-2 tube in BWR-HWC is slightly higher than the oxide formed in BWR-NWC, the weight gain is seen to be higher in BWR-NWC when compared to that in BWR-HWC.

#### 3.2.2. FA-SMT Tube

**Figure 16:**
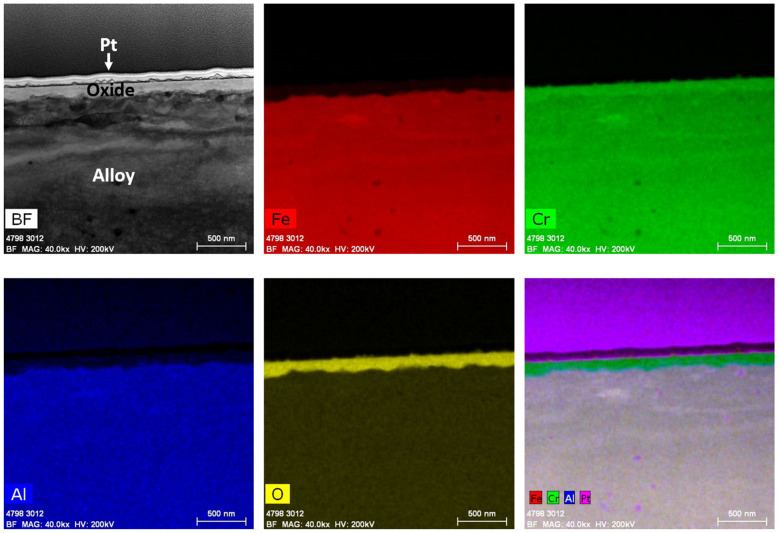
TEM-EDS for FA-SMT (tube) after 6 months of exposure in BWR-HWC (S-13). Marker is 500 nm long.

**Figure 17:**
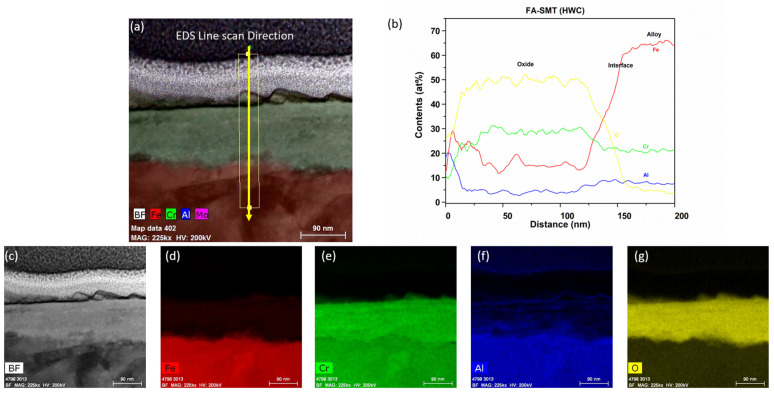
(**a**) shows the EDS line scan direction, (**b**) shows the line scans of the oxide–substrate interfaces in the FA-SMT tube after 6 months of simulated BWR-HWC exposure, and (**c**–**g**) show the high-magnification EDS mapping of the FA-SMT tube.

After six months of immersion testing in BWR-HWC (S-13), a low-magnification TEM-EDS analysis was conducted on the FA-SMT tube, and the results are shown in Figure 16. The TEM bright field images display a ~150 nm thick oxide layer on the FA-SMT tube (Figure 17). The EDS elemental maps confirmed the oxide to be in the form of an FeCr oxide layer. This oxide layer is depleted of iron, indicating the dissolution of Fe under BWR-HWC. At the oxide–alloy interface, a slight enrichment of Al can also be observed via a line scan. After 6 months of immersion testing, the mass loss on the FA-SMT tube specimen is relatively less due to a higher Cr content when compared to PM-C26M (tube) and Fe17Cr5.5Al (flat coupon).

As Cr oxide is stable in this environment, and the coefficient of diffusivity (D_0_) of the Fe cation is higher than that of the Cr cation, the faster Fe cation diffusion can result in mass loss. In the meantime, the formation of a dense and continuous Cr oxide layer may prevent the further diffusion of Fe from the alloy matrix, thus inhibiting the dissolution of Fe from the alloy matrix.

#### 3.2.3. PM-C26M Tube

**Figure 18:**
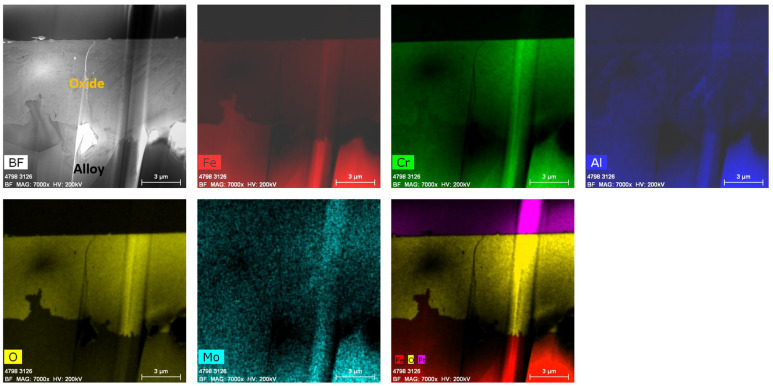
TEM-EDS for PM-C26M (tube) after 6 months of exposure in BWR-HWC (S-13). Marker is 3000 nm long.

**Figure 19:**
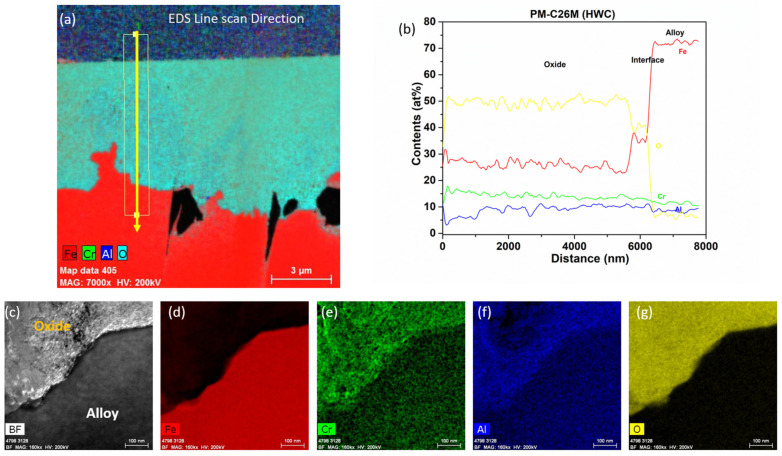
(**a**) shows the EDS line scan direction, (**b**) shows the line scans of the oxide–substrate interfaces in the PM-C26M tube after 6 months of simulated BWR-HWC exposure, and (**c**–**g**) show the high-magnification EDS mapping of the PM-C26M tube.

Figure 18 shows the low-magnification TEM-EDS analysis of PM-C26M (tube) after six months of immersion testing in BWR-HWC (S-13). The bright field images and line scans (Figure 19) confirm the thickness of the oxide developed to be ~8 µm. This thicker oxide layer could be primarily due to a lower Cr content in the alloy matrix. The EDS elemental maps show the external oxide layer to be a mixed Fe-Cr-Al oxide. Even though a similar mass loss was observed on PM-C26M (Tube) and Fe17Cr5.5Al (flat coupons), the thickness of the oxide layer developed on PM-C26M (tube) is twofold higher than the oxide developed on Fe17Cr5.5Al (flat coupons).

#### 3.2.4. Fe17Cr5.5Al Flat Coupon

**Figure 20:**
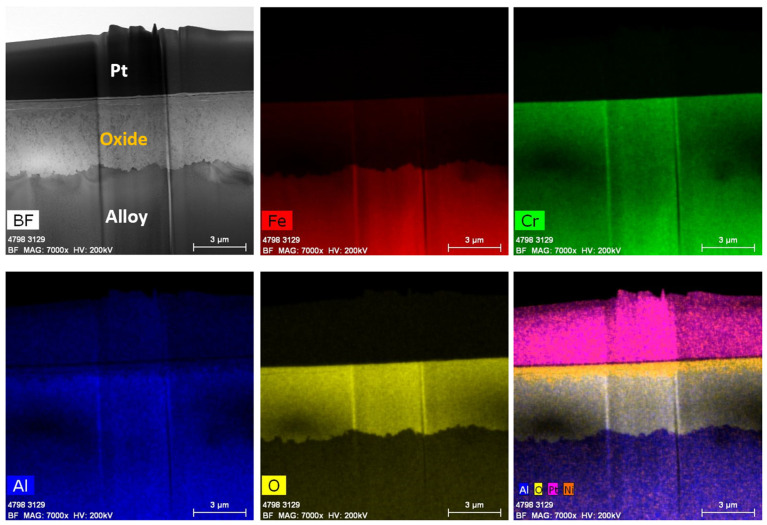
TEM-EDS for Fe17Cr5.5Al flat coupon after 6 months of exposure in BWR-HWC (S-13). Marker is 3000 nm long.

**Figure 21:**
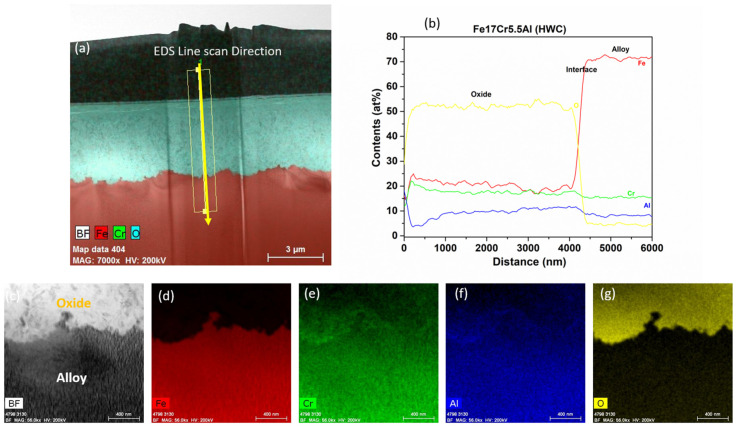
(**a**) shows the EDS line scan direction, (**b**) shows the line scans of the oxide–substrate interfaces in the Fe17Cr5.5Al flat coupon after 6 months of simulated BWR-HWC exposure, and (**c**–**g**) show the high-magnification EDS mapping of the Fe17Cr.5.5Al flat coupon.

Figure 20 shows the low-magnification TEM-EDS analysis of Fe17Cr5.5Al (flat coupons), after six months of immersion testing in BWR-HWC (S-13). The high-magnification bright field image and line scan (Figure 21) confirm the thickness of the oxide developed to be ~4 µm. This thicker oxide layer on the flat coupon could be due to a lower Cr content in the Fe17Cr5.5Al alloy when compared to FA-SMT (tube). The EDS elemental maps showcase a mixed Fe-Cr-Al oxide. The thickness of the oxide developed on Fe17Cr5.5Al (flat coupon) is several orders of magnitude higher than the oxide developed on FA-SMT. It is also observed that a significant mass loss is seen in Fe17Cr5.5Al (flat coupons) due to the continuous oxidation of the metal along with a continuous dissolution of the Fe oxide at the water–oxide interface.

#### 3.2.5. SS316 Tube

**Figure 22:**
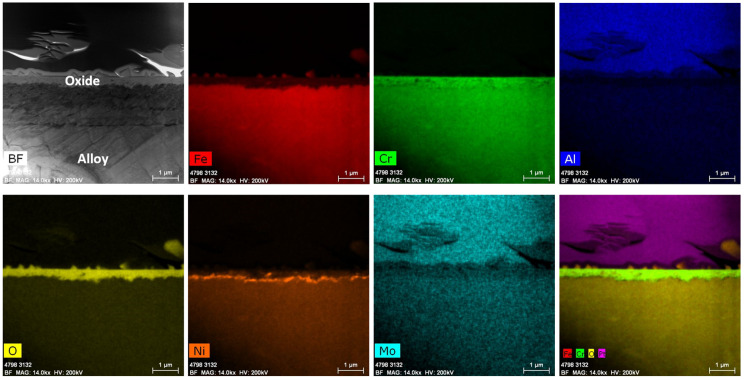
TEM-EDS for SS316 (tube) after 6 months of exposure in BWR-HWC (S-13). Marker is 1000 nm long.

**Figure 23:**
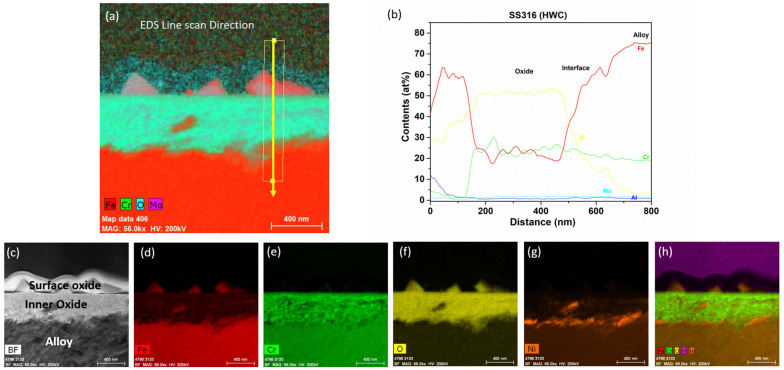
(**a**) shows the EDS line scan direction, (**b**) shows the line scans of the oxide–substrate interfaces in the SS316 tube after 6 months of simulated BWR-HWC exposure, and (**c**–**h**) show the high-magnification EDS mapping of the SS316 tube.

After immersion testing in the BWR-HWC (S-13) for six months, a TEM analysis was conducted on the SS316 tube specimens. The oxide layer formed on the SS316 tube was calculated to be ~500 nm using a low-magnification TEM-EDS analysis (Figure 22). As shown in Figure 23, the high-magnification TEM-EDS elemental maps showcase a surface oxide spinel along with an inner oxide layer at the oxide–alloy matrix interface. The surface oxide mainly consists of Fe spinel and is not continuous, whereas the continuous internal layer is composed of FeCr oxide. Under hydrogenated water, the SS316 tube specimens displayed a lower mass gain than that of the Zirc-2 tube specimens.

### 3.3. Comparison of Oxide Thickness on Alloys as Function of Water Chemistry

**Figure 24:**
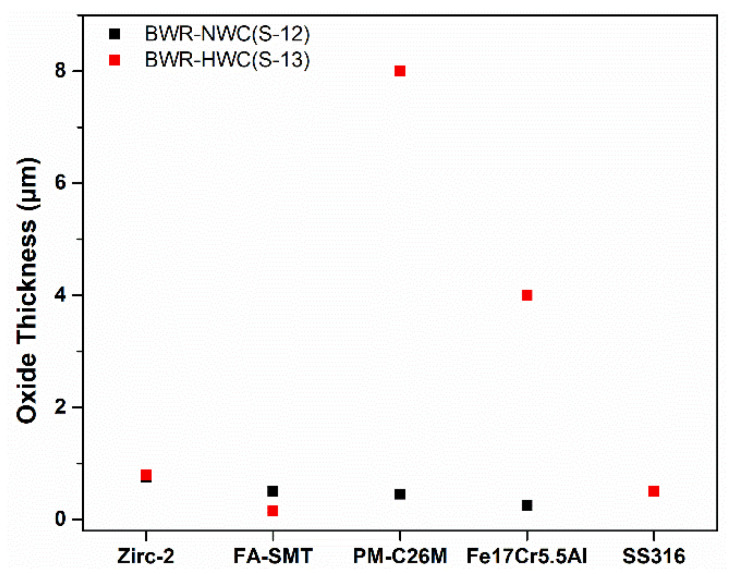
Oxide thickness comparison of all alloy variants tested in simulated BWR-NWC (S-12) and BWR-HWC (S-13) waters for six months.

Based on the data in Section 3.1 and Section 3.2, a box-plot was generated to compare the oxide thickness of all the FeCrAl alloy variants. Figure 24 shows that the oxide thickness developed on the FeCrAl alloy variants was tested in both water chemistries. In oxygenated water, the FeCrAl alloy variants developed thinner oxides. Among the alloy variants, the Fe17Cr5.5Al flat coupon had the thinnest oxide compared to the other FeCrAl alloys, FA-SMT and PM-C26M and the reference materials, Zirc-2 and SS316. However, in hydrogenated water, the oxide that developed on the FA-SMT tube specimen was in nm range, whereas the oxide that developed on PM-C26M and Fe17Cr5.5Al was in the micron range. A trend of increase in the oxide thickness was observed with the decrease in the Cr content and is consistent with Raiman’s observations [10].

## 4. Discussion

The current results show the corrosion behavior of the latest generation of FeCrAl alloys tested in simulated BWR water chemistries for 6 months. In Figure 1, especially for hydrogenated water, the weight loss for all FeCrAl alloy variants is seen and is significantly high for the PM-C26M and Fe17Cr5.5Al specimens. In the case of BWR-NWC, weight gain was observed for the FeCrAl alloy variants and is contradictory with the long-term immersion testing data of the previous generation of FeCrAl alloys developed at ORNL [10] and GE [14,24]. This observation could be due to the lower oxygen concentration (0.5 ppm) used in the current study. Long-term immersion tests need to be conducted to gain further insights into the mass change in these alloys.

**Figure 25:**
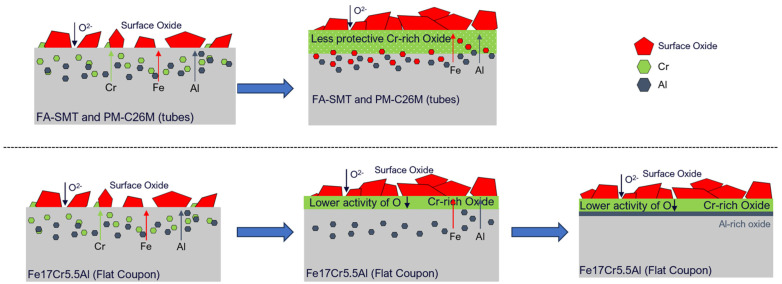
Illustration of oxidation behavior of FeCrAl alloys in simulated BWR-NWC.

After immersion testing in BWR-NWC for six months, a clear enrichment of an FeCr oxide spinel was observed on the FA-SMT specimen. The Cr oxide layer developed underneath the Fe-Cr oxide spinel structure is not continuous and is defective, probably because of the short testing time (Figure 6). On PM-C26M (tube), the Fe-Cr oxide spinel is still growing to be an effective water barrier (Figure 8) so that it can prevent the Cr oxide beneath the spinel from continuously dissolving into the water [25]. This observation can also be attributed to the lower O_2_ concentration in the testing solution (0.5 ppm), which did not provide sufficiently acute oxygen supply for a continuous Cr oxide layer to grow below the Fe-Cr oxide spinel. In the case of the model alloy Fe17Cr5.5Al flat coupon specimens, a surface Fe-Cr oxide spinel was observed, followed by enriched Cr and Al oxides (Figure 10). It is believed that the continuous Cr oxide layer underneath the spinel provides a low partial pressure of oxygen activity [26] that is needed to stabilize Al oxide [27]. While the FA-SMT and PM-C26M tube specimens are fabricated by powder metallurgy, the model alloy flat coupons are manufactured by VIM. Based on our observations, the difference in manufacturing route could be the reason for the enrichment of Al oxide in the Fe17Cr5.5Al flat coupon specimens [18]. Thus, the oxide layer of the model Fe17Cr5.5Al specimen (~200 nm) is much thinner than that of the PM-C26M and FA-SMT (~600 nm) tubes. A summary of the FeCrAl claddings’ oxidation behavior in simulated BWR-NWC is shown in Figure 25.

In the case of BWR-HWC after six months of immersion testing, a trend of increase in the oxide thickness with a decrease in the Cr content was observed (Figure 24). No surface crystals were observed in hydrogenated water compared to oxygenated water, and this finding is consistent with the literature [14]. At 288 °C, even though the thicknesses of the oxides developed on the Fe17Cr5.5Al (flat coupons) (Figure 19) and PM-C26M (tube) (Figure 21) alloys are much larger than that on FA-SMT (tube) (Figure 17), these oxide layers may not form rapid passivation by the Cr oxide due to a smaller Cr content. The fact that Cr oxide is not continuous could be the reason for a huge weight loss of 120 mg/dm^2^ in the alloys containing a lower Cr content after 6 months of exposure, which is consistent with Rebak’s [20] and Raiman’s observations [10]. The literature also hints that the presence of a higher Cr content may possibly slow down the diffusion of other cations [28].

**Figure 26:**
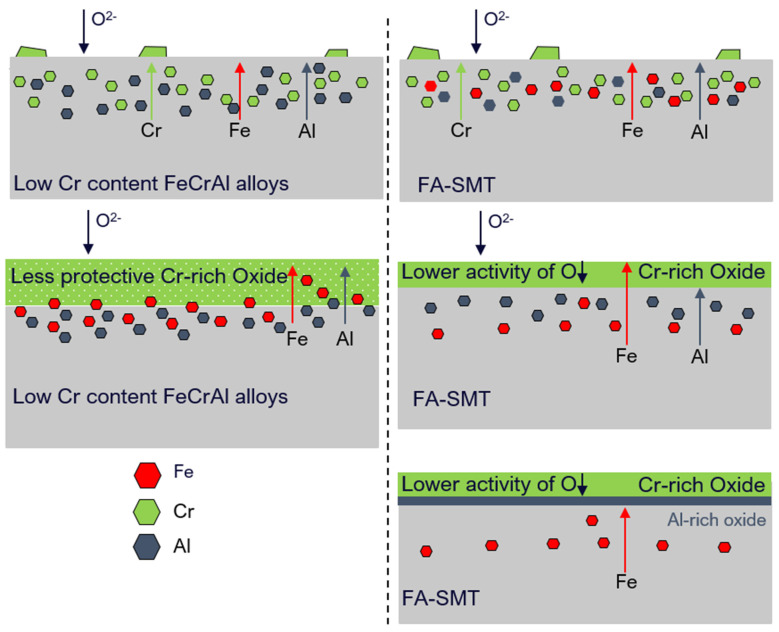
Illustration of oxidation behavior of FeCrAl alloys in simulated BWR-HWC.

The oxygen activity in BWR-HWC is lower due to hydrogen addition [29]. The corrosion with the higher Cr content (FA-SMT tube) may procced by facilitating the outward diffusion of Fe and Cr towards the electrolyte interface, which may enhance the formation kinetics of chromia, thus preventing the dissolved oxygen and hydrogen ingress into the alloy with the high Cr content. At the same time, this continuous protective chromia layer may prevent the further diffusion of Fe from the alloy, thus decreasing the Fe dissolution. Hence, a thinner oxide layer and a lower mass loss is possible, as observed for the FA-SMT tube specimen under this testing condition. Unlike the FA-SMT specimens, the Cr contents in the PM-C26M (tube) and Fe17Cr5.5Al flat coupons are lower. As a result, the chances for the formation of protective chromia are smaller, leading to a continuous dissolution of Fe from the alloys. Hence, a thicker oxide layer and higher mass loss was observed for alloys with lower Cr contents under hydrogenated waters. A summary of the FeCrAl claddings’ oxidation behavior in simulated BWR-HWC is shown in Figure 26.

While all of the FeCrAl alloys displayed a mass loss in BWR-HWC and a slight mass gain in BWR-NWC, the Zirc-2 tubes, irrespective of the test conditions, developed a uniform film of oxide post-test. Due to the formation of this protective oxide in both NWC and HWC, mass gain was observed on the Zirc-2 tubes [10].

## 5. Conclusions

To design and study an FeCrAl alloy composition space that results in next-generation ATF cladding candidates, an attempt was made to study the latest versions of FA-SMT, PM-C26M, and Fe-17Cr-5.5Al specimens in BWR-NWC and BWR-HWC for 6 months. The conclusions from the current study are as follows:

1. All three variants of FeCrAl displayed a weight gain in BWR-NWC. However, in BWR-HWC, all variants displayed a mass loss.
2. For the Fe17Cr5.5Al specimen in simulated BWR-NWC, due to the low partial pressure of oxygen, an Al oxide layer formed beneath the Cr oxide layer.
3. In BWR-HWC, only the FA-SMT specimen displayed a slight enrichment of Al under the protective Cr oxide.
4. Unlike the BWR-NWC specimens, no surface spinel was observed in any FeCrAl alloy variants tested in BWR-HWC.
5. Overall, the latest generation of FeCrAl alloys fabricated via powder metallurgy have little sensitivity to microstructural impact when compared to previous generations of alloys.

## Figures and Tables

**Table 1:** Nominal compositions of FeCrAl alloys studied in this article.

Alloy	Geometry	Chemical Composition in wt%	No. of Samples Tested
FA-SMT	Tube	Fe + 21Cr + 5Al + 3Mo	16
Fe17Cr5.5Al	Flat coupon	Fe + 17Cr + 5.5Al	6
PM-C26M	Tube	Fe + 12Cr + 6Al + 2Mo	16
Zirc-2	Tube	Zr + 1.5Sn + 0.15Fe + 0.1Cr + 0.05Ni	16
SS316	Tube	Fe + 17Cr + 10Ni + 2Mo	16

**Table 2:** Autoclave testing conditions.

Autoclave	Test Conditions, Six-Month Immersion
S-12	Simulated BWR, Normal Water Chemistry (BWR-NWC), 0.5 ppm O_2_, 288 °C
S-13	Simulated BWR, Hydrogen Water Chemistry (BWR-HWC), 0.3 ppm H_2_ (<5 ppb O_2_), 288 °C

## Data Availability

Data are contained within the article.
